# Application of ultrasound in cardiovascular intervention *via* the distal radial artery approach: New wine in old bottles?

**DOI:** 10.3389/fcvm.2022.1019053

**Published:** 2022-12-15

**Authors:** Tao Chen, Xiaolong Yu, Ruixiao Song, Lamei Li, Gaojun Cai

**Affiliations:** ^1^Changzhou Key Laboratory of Molecular Diagnostics and Precision Cancer Medicine, Department of Cardiology, Wujin Institute of Molecular Diagnostics and Precision Cancer Medicine of Jiangsu University, Wujin Hospital Affiliated with Jiangsu University, The Wujin Clinical College of Xuzhou Medical University, Changzhou, Jiangsu, China; ^2^Department of Ultrasonics, Wujin Hospital Affiliated with Jiangsu University, The Wujin Clinical College of Xuzhou Medical University, Changzhou, Jiangsu, China

**Keywords:** ultrasound, distal radial artery, distal radial artery approach, cardiovascular intervention, vascular assessment

## Abstract

The distal radial artery (DRA) approach has emerged as a new approach in cardiovascular intervention. In recent years, ultrasound has been widely used in cardiovascular intervention *via* the DRA approach. This article systematically discusses the progress of ultrasound in the preoperative vascular assessment, intraoperative guided puncture and postoperative observation of complications *via* the DRA approach.

## Introduction

The distal radial artery (DRA) approach has emerged as a new approach in cardiovascular interventions in recent years (1). Compared with the conventional radial artery (CRA) approach, the distal transradial approach (dTRA) dramatically reduces the risk of bleeding and radial artery occlusion (RAO) and offers higher operator and patient comfort (2–5). Although there is no discernible difference between the two techniques in terms of puncture success rate and other characteristics (6), there is a noticeable learning curve for dTRA puncture, with a longer puncture time than the transradial artery approach (TRA) (4, 7).

According to previous studies, puncture of the radial artery under ultrasound guidance can increase cannulation success rates and decrease the risk of haematoma at the puncture site (8–10). Ultrasound is also of high value in cardiovascular interventions performed *via* the dTRA, which can not only improve the success of puncture and catheterization (11–13) and shorten the time of dTRA puncture and catheterization (14) but also monitor vascular access-related problems. In light of the contemporary literature, we systematically evaluated the value of ultrasound in the perioperative period of cardiovascular interventions performed *via* the dTRA.

## Ultrasound examination methods

### Ultrasound images of the anatomical snuffbox

The DRA is divided into the anatomical snuffbox (AS) and the Hegu acupoint by the tendon of the extensor pollicis longus (15). The AS is a triangular depression on the dorsum of the hand near the root of the thumb, with the abductor pollicis longus and extensor pollicis brevis on the outside and the extensor pollicis longus on the inside, the radial styloid process on the lower border, and the trapezium and scaphoid bones at the base (16). The AS contains the DRA, cephalic vein, and superficial branches of the radial nerve (16).

**FIGURE 1:**
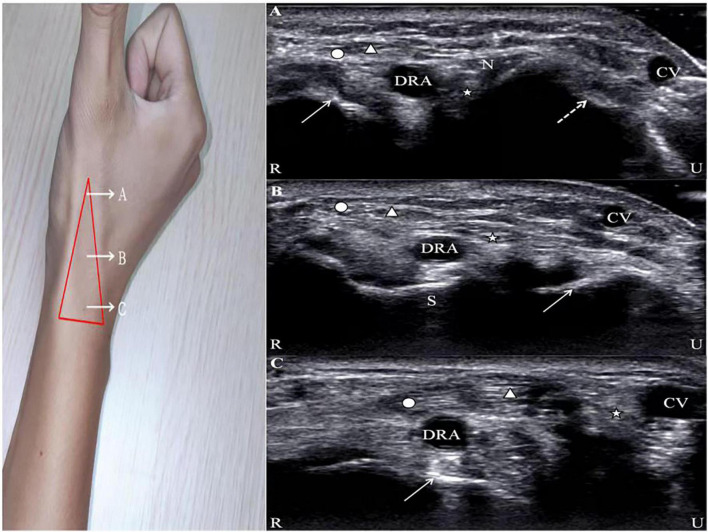
Ultrasound images of the AS. Circle, abductor pollicis longus; Triangle, extensor pollicis brevis; Star, extensor pollicis longus; DRA, distal radial artery; CV, cephalic vein; R, radial; U, ulnar. **(A)** N, radial nerve; solid line arrow, first metacarpal bone; dotted line arrow, second metacarpal bone. **(B)** S, scaphoid; solid line arrow, lunate. **(C)** Solid line arrow, radial styloid process.

The vessel presents a hyperechoic anterior and posterior wall on two-dimensional ultrasound imaging in the long-axis view as well as a long, black, anechoic train-track structure in the middle and an anechoic oval structure in the short-axis view. The superficial branch of the radial nerve is located near the extensor pollicis longus, which presents with small striated structures with hypoechoic and hyperechoic lines alternating on the long-axis view and honeycomb-like structures with a mixture of hypoechoic and hyperechoic lines and annular hyperechoic wrapping on the short-axis view. Similar to the radial nerve, the image of the tendon displays a striatal-like structure along the long axis and a compact, fibrillar highly echogenic elliptical structure along the short axis with a terminal attachment to the bone. The bone cortical surface shows a strong echogenic signal with a posterior acoustic shadow (17) (Figure 1).

### Ultrasound operation in the anatomical snuffbox

**FIGURE 2:**
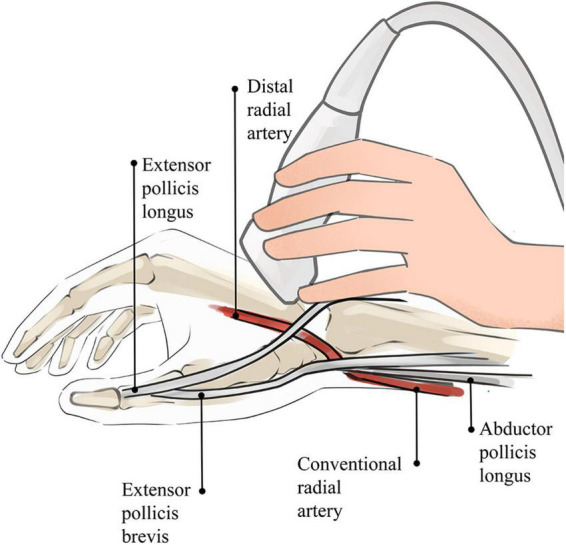
Sketch of ultrasound technique of AS. The operator’s little finger touches the patient’s forearm as a support point, which prevents the blood vessel from being affected by external forces.

When the ultrasound examination is performed in the AS, the patient’s hand is placed in a relaxed position, as in holding a wine glass, to fully expose the AS region. After applying the proper amount of ultrasound coupling agent, the thumb, index finger, and middle finger of the operator are used to secure the ultrasound probe, with the little finger touching the patient’s forearm as a support point (Figure 2). As the DRA is usually superficial, excessive pressure is likely to cause measurement errors; thus, the ultrasound probe is placed lightly perpendicular to the skin until it contacts the coupling agent. A high-frequency (6–18 MHz) linear probe is usually chosen, and its size is appropriate to place in the narrow AS region (18).

### Ultrasound-guided arterial puncture method

**FIGURE 3:**
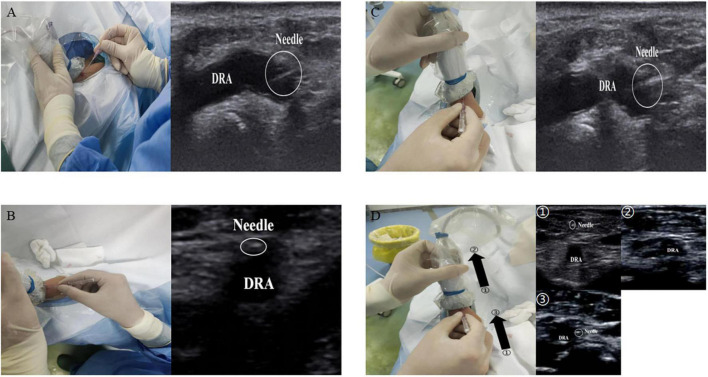
Ultrasound guided puncture. **(A)** The long-axis in-plane method; **(B)** the short-axis out-of-plane method; **(C)** the oblique axis in-plane method; **(D)** the dynamic needle tip positioning. ➀, seeing the highlighted needle tip; ➁, holding the puncture needle still and translating the probe proximally until the tip disappears; ➂, followed by holding the ultrasound probe still and advancing the puncture needle until the tip reappears in the center of the vessel.

Ultrasound guidance can significantly improve the success rate of radial artery puncture (11). The basic methods for performing ultrasound-guided arterial puncture include the long-axis in-plane method, the short-axis out-of-plane method, the oblique axis in-plane method, and dynamic needle tip positioning (19, 20). In the long-axis in-plane method, the long-axis of the ultrasound probe is parallel to the longitudinal axis of the vessel, which appears as a tubular hypoechoic structure on imaging. During vascular puncture, the needle is inserted at 15°–30° to the skin with the tip parallel to the long axis of the vessel. The short-axis out-of-plane method involves placing the ultrasound probe perpendicular to the direction of the vessel, which then appears as a circular hypoechoic structure on imaging. The puncture needle is also inserted at 15°–30° to the skin with the tip perpendicular to the short-axis of the vessel. The oblique in-plane method is based on the short-axis out-of-plane method with an additional 45° clockwise rotation of the probe; the resulting vascular image is an elliptical hypoechoic structure. Dynamic needle tip positioning involves seeing the highlighted needle tip on the ultrasound image, holding the puncture needle still and translating the probe proximally until the tip disappears, followed by holding the ultrasound probe still and advancing the puncture needle until the tip reappears in the center of the vessel (Figure 3). Meta-analyses have revealed no significant difference among the various puncture guidance techniques for increasing radial artery puncture success rates (21, 22). Until now, there have been no studies on whether various ultrasonography manoeuvres are helpful in increasing the success rate of puncture *via* the dTRA.

## Application of ultrasound in distal radial artery interventions

Measurement of the vascular diameter before surgery, puncture guidance during catheterization, and evaluation of complications related to puncture after surgery are of high clinical value. Currently, there are a large number of studies on the use of ultrasound for cardiovascular intervention *via* the DRA. We systematically searched the relevant literature in the PubMed database using the keywords “distal radial artery, distal radial approach, distal radial access, distal transradial artery, distal transradial approach, distal transradial access” and “ultrasound, ultrasound-guided, ultrasonography, duplex ultrasound” (last search date 2022.07.10), combined with a manual search of the references of the retrieved articles. A total of 3,581 articles were initially retrieved. Based on the titles and abstracts and removing duplicate articles, a total of 22 studies were ultimately enrolled (2, 6, 12, 13, 23–40), with thirteen studies from Asia, five from Europe, two from North America, and two from South America. The sample sizes ranged from 42 to 2,775 cases. The study types included two randomized controlled studies, six prospective cohort studies, one cross-sectional study, and thirteen retrospective studies (Table 1).

**TABLE 1:** DRA diameter and the influencing factors.

References	Country	Study design	Number (*n*)	Ultrasound machine model	Probe type	Probe frequency	Diameter definition	Diameter of distal radial artery (mm)	Influencing factors
Kaledin et al. (38)	Russia	Prospective	2,775	NA	NA	NA	Media to media	2.4*	NA
Kim et al. (31)	Korea	Retrospective	117	NA	NA	NA	Media to media	2.65 ± 0.46 men 2.40 ± 0.53 women	Gender
Lee et al. (32)	Korea	Prospective	151	NA	NA	NA	Adventitia to adventitia	2.41 ± 0.50 L 2.36 ± 0.49 R	NA
Babunashvili (39)	Russia	Retrospective	1631	NA	NA	NA	Media to media	2.13 ± 0.33	NA
Flores (40)	America	Retrospective	200	NA	NA	NA	Media to media	2.4 men^#^ 2.1 women^#^	NA
Norimatsu et al. (24)	Japan	Retrospective	142	NA	NA	NA	Adventitia to adventitia	2.60 ± 0.50	Weight, CRA, BMI
Yu et al. (25)	China	Retrospective	92	Philips, iE33	Philips L11-3	NA	Media to media	1.70 ± 0.50 L 1.71 ± 0.50 R	NA
Mizuguchi et al. (28)	Japan	Retrospective	228	Boston scientific corporation	Linear probe	11 MHz	Media to media	2.40 ± 0.50 AS 2.30 ± 0.50 Hegu	NA
Naito et al. (29)	Japan	Retrospective	120	Philips, CX50	Linear probe	12–13 MHz	Media to media	2.02 ± 0.44	NA
Wang et al. (6)	China	Retrospective	620	NA	NA	NA	Media to media	2.20 ± 0.50	NA
Hadjivassiliou et al. (12)	Canada	Retrospective	287	NA	NA	NA	Media to media	2.34 ± 0.36	NA
Kawamura et al. (30)	Japan	Retrospective	50	NA	NA	NA	Media to media	2.99 ± 0.60	NA
Meo et al. (34)	Italy	Cross-sectional	700	Esaote MyLab two, Genova	Linear probe	10 MHz	Media to media	1.99 ± 0.47	Gender, BMI
Koury et al. (35)	Brazil	Prospective	42	NA	NA	NA	Media to media	2.31 ± 0.44	NA
Achim et al. (2)	Romania	Prospective	1240	NA	Linear probe	7.5 MHz	Media to media	2.30 ± 0.20	NA
Lee et al. (33)	Korea	Retrospective	1162	Vivid E95/Philips EPIQ7	11L-D/L12-3 linear probe	4.5–12 MHz /3–12 MHz	Media to media	2.35 ± 0.45 L 2.31 ± 0.43 R	Gender, BMI, BSA
Eid-Lidt et al. (37)	Mexico	Randomized	140	L741	SonoScapeE2	16–4 MHz	Media to media	2.40 ± 0.50	NA
Ghose et al. (13)	India	Prospective	108	NA	Philips, M2540 A	NA	Media to media	2.25 ± 0.34	NA
Deora et al. (23)	India	Prospective	1004	Philips, EPIC 7C	NA	8–11 MHz	Media to media	2.23 ± 0.39	Height, weight, gender
Li et al. (26)	China	Retrospective	246	NA	Philips, EPIQ 7C/IE33	NA	Media to media	2.05 ± 0.41	Gender
Xiong et al. (27)	China	Randomized	81	Wisonic, China	Linear probe	4–15 MHz	Media to media	2.10 ± 0.40	Weight
Horák et al. (36)	Czech Republic	Retrospective	115	GE Vivid S6	Linear 9L transducer	2.4–10.0 MHz	Media to media	2.31 ± 0.47	NA

*median values; ^#^mean; L, Left; R, Right; NA, non-available; CRA, conventional radial artery; BMI, body mass index; BSA, body surface area; AS, anatomical snuffbox; Hegu, Hegu acupoint.

### Preoperative measurement of vessel diameter

**FIGURE 4:**
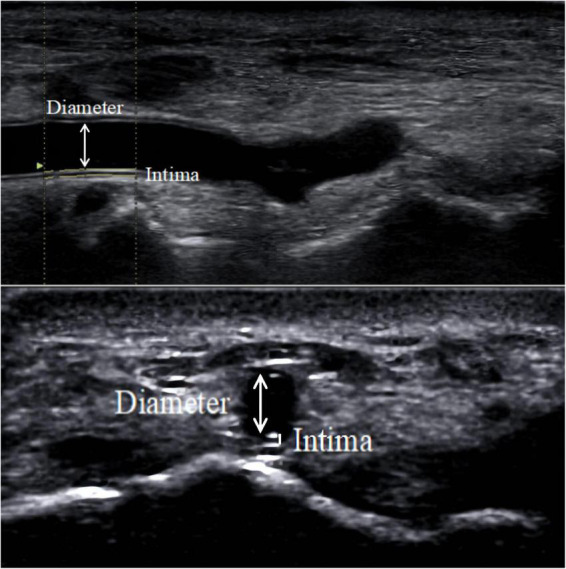
DRA diameter. The diameter measured in the figure is the distance from the media to the media.

The results of a meta-analysis showed that the size of the outer diameter of the vascular sheath was positively correlated with the incidence of RAO (41), and mismatch between the sheath and vessel diameter increased the incidence of RAO. To reduce the risk of vascular injury, an increasing number of studies have focused on the preoperative assessment of the DRA diameter (2, 6, 12, 13, 23–40). Selection of suitable sheaths according to the diameter of the DRA appeared to reduce vascular damage (24). Although computed tomography or angiography can accurately measure vessel diameter (42–44), radiation and contrast agents are required and expensive. In contrast, ultrasound is a convenient and non-invasive method for evaluating vessel diameter (Figure 4).

#### Distal radial artery diameter and the influencing factors

As shown in Table 1, the diameter of the DRA varied greatly in various studies, which may be related to race, characteristics of the research objects, measurement location, diameter definition, demographic morphological characteristics, etc. (24, 25, 28–30, 37). As reported by Yu WW et al., the DRA diameter was 1.71 ± 0.50 mm in a Chinese population (25), but 2.40 ± 0.50 mm according to data from Mexico (37). The largest DRA diameter measured to date is 2.99 ± 0.60 mm in Japan (30) from a study that excluded subjects with a vessel diameter < 2.0 mm. In another Japanese population, Naito T et al. showed a DRA diameter of 2.02 ± 0.44 mm (29). According to Meo D et al., the diameter of the DRA was significantly larger in men than in women (34). Additionally, for the DRA, differences in vessel diameters measured inside and outside the AS have been found. The diameter was smaller at the Hegu acupoint than at the AS (28). In addition, the methods used to measure the vessel diameter were different. In most studies, the definition of vessel diameter was the media to media distance (25, 27, 29), while the distance from the outer membrane to the outer membrane was used in other studies (24, 32). For example, Norimatsu K found the diameter of the DRA, defined as the distance from the outer membrane to the outer membrane, to be 2.6 ± 0.5 mm, which was larger than the results of other studies (24). A similar result was obtained in a Korean population (32).

The DRA is the continuation of the CRA on the back of the hand. Does the diameter of the CRA affect the diameter of the DRA? With the exception of one Canadian study (44), the majority of studies discovered a correlation between DRA and CRA diameters, with a ratio of almost 80% (24, 29, 33). Norimatsu K et al. highlighted a strong positive correlation (*r* = 0.66, *P* < 0.0001) between the DRA diameter to the CRA diameter, and the ratio of the DRA to CRA diameter was 0.8 ± 0.1 (24). Naito T et al. also found a strong correlation between the DRA and CRA diameters (29). In a large-sample Korean study, the DRA/CRA ratio was similar to the previous results on both sides (33). However, in another study, the DRA diameter was 2.10 ± 0.40 mm, and the CRA diameter was 2.60 ± 0.40 mm, which were not significantly correlated (*r* = 0.053, *P* = 0.641) (27). The diameter of the DRA was even larger than that of the CRA in 6.6–8% of patients (29, 34), considering vascular variation. Most studies have suggested that the CRA diameter affects the DRA diameter.

Sex may be a factor influencing DRA diameter, with studies from Asia, Europe, and the Americas showing that the DRA diameters in females are significantly smaller than those in males (23, 26, 31, 33, 34, 40). However, Norimatsu K et al. found that although the DRA diameter in women was smaller than that in men, the difference was not significant (men: 2.6 mm, women: 2.5 mm, *P* = 0.08) (24). A similar conclusion was also obtained in a study by Horák D et al. (Men: 2.32 mm, Women: 2.25 mm, *P* = 0.27) (36). Despite the conflicting findings, generally, the DRA diameter is larger in men than in women.

The demographic and morphological characteristics of the patients may also influence the diameter of the DRA. Studies have shown that patients with diabetes or hypertension have smaller DRA diameters (2.18 ± 0.41 mm for hypertension, 2.26 ± 0.38 mm for non-hypertension, *P* < 0.001; 2.18 ± 0.44 mm for diabetes, 2.26 ± 0.38 mm for non-diabetes, *P* = 0.004) (23). In 2019, Japanese scholars found a positive correlation between body mass index (BMI) and DRA diameter (*r* = 0.228, *P* = 0.007) (24). Subsequently, a study by Meo D et al. found that subjects with BMI > 30 kg/m^2^ had larger vessel diameters than those with BMI < 30 kg/m^2^ (2.20 ± 0.50 mm vs. 1.97 ± 0.52 mm, *P* < 0.001) (34). Lee WJ et al. showed that low BMI and low body surface area were strong independent predictors of DRA diameter < 2.3 mm (33). In addition, DRA diameter was also reported to be positively correlated with both height and weight (23, 24, 27). In contrast, Naito T et al. discovered no correlation between DRA diameter and patient parameters such height, weight, and BMI (29). In conclusion, demographic and morphological characteristics such as body surface area and the presence of diabetes or hypertension can affect DRA diameter. Due to differences in study design and measurement methods, whether other patient parameters have an effect on DRA diameter has yet to be verified.

#### Effect of endovascular diameter on intraoperative selection of vascular sheath

Sheath placement may cause damage to the DRA (24). Hadjivassiliou A et al. concluded that different types of sheaths can be safely placed within different sized DRA (1.8–2.8 mm in diameter) (12). However, a retrospective study reported that an artery/sheath ratio > 1.0 was least damaging to the vessel during coronary intervention *via* the DRA (29). With conventional sheaths (Super Sheath™), the percentages of artery/sheath ratios ≥ 1.0 were 94, 77, 55, and 34% for 4 Fr (1.88 mm), 5 Fr (2.2 mm), 6 Fr (2.5 m), and 7 Fr (2.84 mm), respectively. In contrast, when using a slender sheath (Glidesheath Slender^®^), the percentages of artery/sheath ratios ≥ 1.0 were 81, 63, and 39% for 5 Fr (2.13 mm), 6 Fr (2.46 mm), and 7 Fr (2.79 mm), respectively (24). Only 57.6% of patients were suitable for placement of a 5 Fr catheter sheath (2.3 mm), and 35.1% were suitable for placement of a 6 Fr catheter sheath (2.7 mm) in Korean patients (32). This is similar to results obtained in European countries (34). The use of preoperative ultrasound to examine vessel size is an important guide for the selection of sheaths, and the use of slender or sheathless techniques may reduce vascular-related complications (33, 45).

#### Postoperative diameter change

The cannulation procedure may cause changes in the DRA vessel diameter (28, 30). Mizuguchi Y et al. used ultrasound to measure the DRA diameter before and after the procedure and observed a dynamic change in vessel diameter, which was 2.40 ± 0.50 mm before the operation, expanded significantly to 2.70 ± 0.50 mm 1 day after the operation and returned to 2.50 ± 0.50 mm 1 month after the operation (28). Similar vascular changes were observed in another study (30).

### Operation guidance

#### Ultrasound guidance increases the success rate of distal radial artery puncture

Traditionally, interventionalists utilize palpation to identify the puncture site when performing radial artery puncture. However, pulse palpation in the DRA can be challenging and difficult in some conditions. For example, pulsation is weak in patients with multiple punctures, patients suffering from shock or peripheral vascular disease, and patients with RAO, who have only 70% arterial pressure on average at the distal remnant of the occluded radial artery (46). Additionally, in patients with small vessel diameters, it is difficult to identify the course of the blood vessels. These conditions make the patient prone to puncture failure. However, if ultrasound is used, successful puncture of the DRA may be obtained with ultrasound guidance even if the pulse is weak or the vessel diameter is small (47). Ultrasound-guided DRA cannulation can be used not only for coronary interventions but also for abdominopelvic and neurological interventions (35, 48). This visualization of the puncture not only increases the success rate but also reduces the complications, especially for precise or emergency interventions.

In recent years, an increasing number of studies have explored the relationship between ultrasound guidance and puncture success rate (11–13). Although in a small sample retrospective cohort study, the puncture success rate was similar between ultrasound-guided and palpation methods (49). In general, the success rate of ultrasound-guided puncture is higher than that of conventional dTRA puncture (11). A study that included 108 individuals demonstrated a high success rate from ultrasound-guided puncture (96.3%) as well as a reduced incidence of DRA spasm (13). As reported by Hadjivassiliou A et al., the puncture success rate in the left DRA reached 100% under ultrasound guidance (12). Possible biases in the conclusions were generally due to the small sample size, and the majority of research still favours ultrasound guidance to increase puncture success. Nevertheless, Tsigkas G et al. emphasized that although ultrasound guidance could improve the success rate of puncture, it might increase the procedure time and cost, and it was not advocated as a routine operation (3). Moreover, ultrasound guidance has a high value in the reverse recanalization of the proximal RAO *via* the DRA (50). In RAO patients, the radial artery in the AS circulates through the reverse lateral branch of the palmar arch. The palpation becomes weak or disappears, but blood flow signals are still visible under high-frequency ultrasound. Ultrasound guidance may significantly increase the puncture success rate (50, 51).

#### Ultrasound guidance reduces operation complications

During puncture, ultrasound can be used to identify the anatomical structures around the blood vessels and avoid damage to normal tissue as much as possible (40). Rapid resolution of the arteriovenous system by means of a “compressibility test” with a probe showed pulsation of the artery, which did not collapse completely under pressure, and the vein was easily deflated completely. Ultrasound can also identify shallow radial nerves to avoid potential nerve damage (18). However, previous studies have also found no significant difference in vascular access-related complications (haematoma and neuropathy) with or without ultrasound guidance (11).

### Evaluation of complications

**FIGURE 5:**
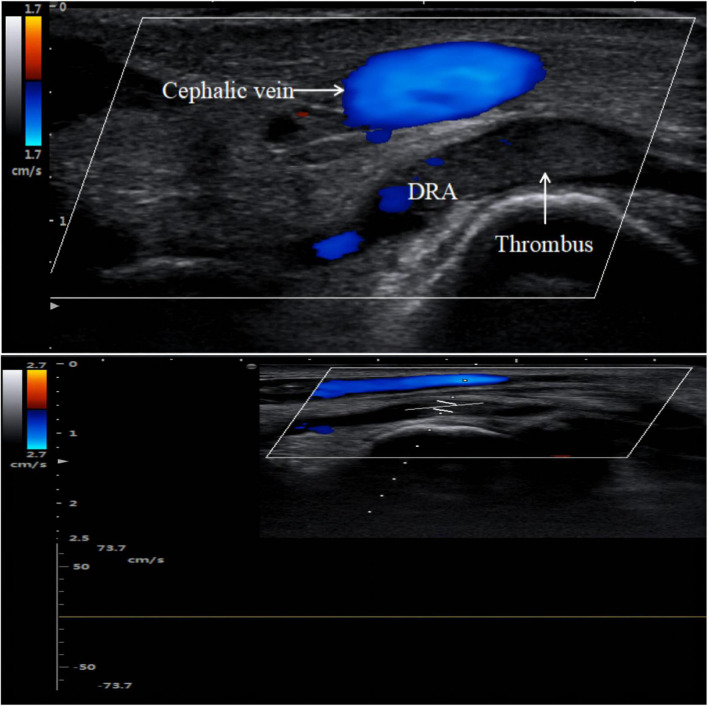
Distal radial artery occlusion. Thrombus is hypoechoic on color Doppler imaging. There is no blood flow spectrum waveform curve on pulse wave Doppler imaging.

Radial artery occlusion is a common complication of TRA interventions in clinical practice. The incidence of RAO after the TRA has been reported to range from 2.5 to 8.4%, which is higher than that after the dTRA (0–5%) (4, 45). In the past, some methods, such as palpation, the reverse Allen test, and the reverse Barbeau test, were typically used to determine the presence of RAO (52, 53). Due to the reverse blood supply of the collateral circulation and the fact that most patients with RAO are asymptomatic, only a small percentage of patients show hand ischaemia (54). The methods listed above for detecting RAO have the potential for false negatives, which can easily lead to missing a diagnosis of RAO. However, the absence of a blood flow signal in the occluded radial artery can be observed by ultrasound (Figure 5), which can greatly reduce the rate of underdiagnosis of RAO (6, 36). Complications such as distal radial artery occlusion, haematoma, bleeding, arteriovenous fistula, pseudoaneurysm, infection, and entrapment are equally common after coronary intervention *via* the dTRA (38, 55, 56). In recent years, the incidence of distal radial artery occlusion after dTRA intervention has been reported to range from 0.12 to 5.2% (28, 38–40, 57). In 2014, Kaledin A et al. reported a 2.2% incidence of RAO in 1,009 patients after dTRA, with 0.1% occlusion in the forearm, 1.8% in the AS, and 0.3% in both the forearm and AS (38). Subsequently, Mizuguchi Y et al. showed that the incidence of distal radial artery occlusion was 3.5% (8 cases/228 cases) in a Japanese population (28).

In addition, ultrasound follow-up revealed thrombus autolysis after acute RAO. The incidence of RAO was 8.4% at 24 h after intervention *via* CRA and decreased to 5.6% after 30 days, with a recanalization rate as high as 33.3% (37). In a meta-analysis, the incidence of RAO was 7.7% for the TRA at 24 h postoperatively and decreased to 5.8% at 30 days (41). Ultrasound can be performed repeatedly and has unique advantages in determining whether the radial artery is recanalized at follow-up.

Although the radial artery was not occluded in some patients after transradial catheterization, intravascular ultrasound revealed thickening of the CRA intima (intima-media thickness > 0.4 mm), resulting in stenosis (58). Intimal changes after radial artery cannulation were observed by optical coherence tomography, which also revealed postoperative CRA intimal thickening (59), and a significant increase in CRA intimal volume was still seen at the 9-month postoperative follow-up (60). Despite the fact that optical coherence tomography and intravascular ultrasound are more reliable at detecting endovascular injury, they are also more invasive and expensive, involve limited exploration of the vessels and should not be utilized as standard diagnostics. In addition to being non-invasive, ultrasound also offers an infinite range of detection and can be utilized as part of a standard postoperative assessment of the vessel intima (61, 62). Thus, ultrasound plays a major role in detecting the histopathological morphology and structure of radial artery vessels.

Furthermore, ultrasound can measure radial artery haemodynamic changes before and after the operation. Peak systolic velocity (PSV) in the radial artery was measured using ultrasound at different times after surgery. The preoperative PSV was 59.6 cm/s, which significantly increased to 63.1 cm/s half an hour later and decreased back to 57.3 cm/s 7 days after the operation (63). A study simulating RAO demonstrated that the PSV in the forearm radial artery and the radio-dorsal digital artery of the thumb were significantly reduced in the presence of RAO at the wrist level (forearm radial artery: 24 ± 13% of baseline, *P* = 0.001; digital artery of the thumb: 68 ± 8% of baseline, *P* = 0.001); however, the PSV in the forearm radial artery and the digital artery of the thumb were essentially unchanged when RAO was performed at the AS level (forearm radial artery: 93 ± 2% of baseline, *P* = 0.65; digital artery of the thumb: 95 ± 2% of baseline, *P* = 0.71) (64).

Ultrasound also helps to improve the efficiency of treatment for postoperative complications. For postoperative abnormal bulges near the puncture site that are difficult to identify on routine physical examination, such as haematomas, arteriovenous fistulas, or pseudoaneurysms, ultrasound can clarify the diagnosis. In patients with arteriovenous fistulas, ultrasound can probe the vascular breach for applying precise compression bandaging treatment to reduce patient pain (55, 65). Additionally, patients with pseudoaneurysms can be treated not only with ultrasound-guided compression bandaging (56, 66) but also with ultrasound-guided injection of thrombin (67).

## Limitations

**FIGURE 6:**
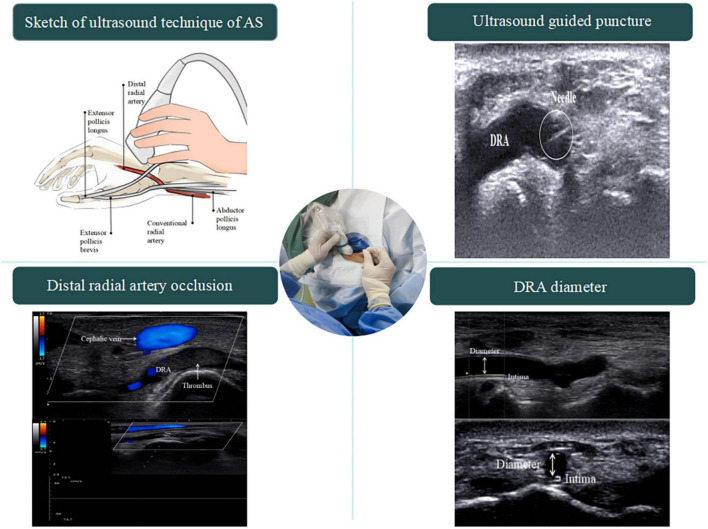
Central illustration.

Interventional methods *via* DRA are only now emerging. The application of ultrasound has been recommended by some scholars (Figure 6). However, due to the special anatomical location of the AS, such as the abundant bony structures and the narrow range (15), it is not easy for the ultrasound probe to fully contact the examination site, which increases the difficulty of ultrasound examination. Moreover, unskilled operators require a learning curve (7), which may prolong the procedure time. Therefore, ultrasound guidance is difficult to apply in high-volume centers. The main disadvantage of ultrasound is the lack of a uniform definition and resolution of the machine or the measurement technique, which will influence the measurement results. To adapt to the anatomical characteristics of the AS, a smaller probe needs to be designed to achieve flexible exploration of the target vessels.

In conclusion, ultrasound has certain clinical application value in the process of DRA intervention. In the near future, as medical technology continues to be improved, the application of ultrasound in the interventional treatment of DRA may become more widespread.

## Author contributions

GC conceived and designed the article. XY and RS reviewed the ultrasound diagnosis. LL analyzed the data. TC collected the literature and wrote the manuscript. All authors contributed to the article and approved the submitted version.
